# Assessing the Concurrent Validity of the Australian Treatment Outcomes Profile in a Methamphetamine Dependent Treatment‐Seeking Population

**DOI:** 10.1111/dar.70224

**Published:** 2026-08-10

**Authors:** Brendan Clifford, Rachel M. Deacon, Kristie Mammen, Krista J. Siefried, Nadine Ezard, Nicholas Lintzeris

**Affiliations:** ^1^ Alcohol and Drug Service St Vincent's Hospital Sydney Sydney Australia; ^2^ National Centre for Clinical Research on Emerging Drugs UNSW Sydney Sydney Australia; ^3^ New South Wales Drug and Alcohol Clinical Research and Improvement Network (DACRIN) Sydney Australia; ^4^ National Drug and Alcohol Research Centre UNSW Sydney Sydney Australia; ^5^ Specialty of Addiction Medicine, Faculty of Medicine and Health The University of Sydney Sydney Australia; ^6^ Drug and Alcohol Services South Eastern Sydney Local Health District Sydney Australia; ^7^ Centre for Alcohol and Other Drugs NSW Ministry of Health Sydney Australia

**Keywords:** amphetamine‐related disorders, methamphetamine, outcome assessment, patient reported outcome measures, substance use

## Abstract

**Introduction:**

The Australian Treatment Outcomes Profile (ATOP) is a brief clinical tool assessing substance use, health and well‐being used in Australian alcohol and other drug treatment services. It is validated for use with clients using alcohol, opioids and cannabis, but not yet for clients who primarily use methamphetamine.

**Methods:**

An embedded validation study was undertaken in treatment‐seeking adults enrolled in a randomised double‐blind placebo‐controlled trial of lisdexamfetamine for methamphetamine dependence with sites in New South Wales, South Australia and Victoria. Participant demographics were collected during study screening. The ATOP and comparators (Time Line Follow Back, Opiate Treatment Index, Depression Anxiety Stress Scale, WHOQOL‐BREF and Personal Wellbeing Index) were collected at baseline. Continuous ATOP items were analysed using Pearson's correlation coefficient, and dichotomous items were analysed using Fleiss's *κ*. Agreement was rated as strong where measures were ≥ 0.50, moderate where agreement was 0.30–0.49, and weak where < 0.30.

**Results:**

One hundred and eighteen study participants (2018–2020) had data for concurrent validity analysis. Strong validity was demonstrated for physical health, psychological health, quality of life, injecting drug use and crime items, and for days of use for amphetamines, alcohol, cannabis and cocaine. There was weak validity for days of use for benzodiazepines. Heroin use days and other opioid use days were endorsed by fewer than five participants and were therefore unable to be assessed.

**Discussion and Conclusions:**

The ATOP is valid for use in a treatment‐seeking methamphetamine‐dependent population, expanding the range of tools for assessment and standardised outcome monitoring across different settings and services.

## Key Points

- The Australian Treatment Outcomes Profile (ATOP) is widely used in clinical settings in Australia for assessment, treatment monitoring and outcome reporting.
- The ATOP has been validated in populations seeking treatment for opioid, cannabis and alcohol dependence.
- It has not previously been validated in people seeking treatment for methamphetamine dependence.
- This study validates the ATOP in an Australian methamphetamine treatment‐seeking population.
- The ATOP can be used with confidence in methamphetamine treatment‐seeking populations.

## 1 Introduction

The Australian Treatment Outcomes Profile (ATOP) is an instrument used to assess and monitor treatment outcomes of alcohol and other drug service clients [1]. It can also be used for research and the evaluation of services or interventions [2, 3]. The ATOP was adapted from the Treatment Outcome Profile used in the United Kingdom [4] to be a brief instrument for assessing substance use, health and well‐being over a 4‐week period. It has been validated for client groups seeking treatment for opioid, alcohol and cannabis dependence [5, 6, 7], but not yet for people seeking treatment for methamphetamine dependence. Methamphetamine is an increasingly common substance in Australia for which people seek treatment, with a doubling of treatment episodes over the past decade [8]. In prior evaluations of the ATOP [5, 6, 7], relatively few numbers of enrolled participants reported using amphetamine‐type stimulants, and hence validation of the ATOP items in this client group is required. A validation study was therefore undertaken using data obtained in the LiMA study, a randomised double‐blind placebo‐controlled study of lisdexamfetamine for the treatment of methamphetamine dependence [7].

## 2 Methods

The LiMA study was a fixed‐dose parallel design comparing the efficacy and safety of an outpatient 12‐week maintenance course of oral lisdexamfetamine (250 mg/day) to placebo. Participants were recruited via specialist alcohol and other drug services in New South Wales, South Australia and Victoria [9]. The study population consisted of treatment‐seeking adults with long‐standing (≥ 12 months) methamphetamine dependence, reporting at least 14 days of methamphetamine use in the previous 28 days. Full eligibility details for the study have been previously reported [10], but in summary, participants had to meet ICD‐10 criteria for methamphetamine dependence, self‐report methamphetamine use of 14 or more days out of the previous 28; and have one urine drug screen positive for methamphetamine. They were ineligible if they were receiving opioid agonist therapy; had prescribed stimulants in the previous 4 weeks; or were dependent on alcohol or other non‐prescribed substances. A battery of measures was used at screening, assessment and throughout participation to assess participants' physical health, mental health, drug use and cost‐effectiveness of the intervention [9]. Participants with data for the ATOP, demographic measures and comparator measures outlined below were included in this analysis. LiMA participants enrolled after March 2020 were not included in this analysis as COVID‐19 pandemic adaptations required the omission of some instruments to reduce the time taken for clinic visits. Ethics approval was gained through the St Vincent's Hospital Human Research Ethics Committee (approval number HREC/16/SVH/222).

### 2.1 Measures

Demographic information (age, gender, sexuality, country of birth, Aboriginal or Torres Strait Islander status, highest education level attained, current employment, housing) was collected at screening. Other measures were collected at the baseline visit where participants were inducted onto the investigational medicinal product.

### 2.2 ATOP

The ATOP documents client‐reported responses across a range of key outcome domains in the preceding 28 days [11]. Substance use items are days of use of alcohol, cannabis, heroin, other opioids, benzodiazepines, amphetamine‐like stimulants and cocaine; daily tobacco use; drug injection and injection with equipment used by someone else. Health and well‐being items are days of paid work and study, homelessness, risk of eviction, primary caregiver for or living with, children aged under 5 and 5–15 years, any arrests and if the participant has experienced or perpetrated violence. Clients also rate their overall physical health, psychological health and quality of life on three separate Likert scales from 0 (*poor*) to 10 (*good*).

### 2.3 Gold Standard Comparators

#### 2.3.1 Substance Use

The Timeline Followback technique [12] was used to assess days of use for all substances over the past 28 days, using a calendar and prompts for significant events to improve recall. The TLFB has well‐established high reliability and validity for measuring substance use [13]. The TLFB was used for comparison to ATOP substance use items.

#### 2.3.2 Injecting and Injection With Used Equipment

The Opiate Treatment Index (OTI) is a multidimensional instrument developed to assess clinical outcomes in opioid treatment [14]. The HIV risk drug use sub‐scale (six questions; referring to past month) was used as the comparator to the ATOP injecting days and injection with equipment used by someone else items.

#### 2.3.3 Psychological Health

Two comparators were used to compare to the ATOP psychological health item. The Depression Anxiety Stress Scale is a 21‐item questionnaire rating depression, anxiety and stress in the previous 4 weeks [15]. The total DASS score, a measure of overall psychological distress, was used to compare to the ATOP psychological health item score. The World Health Organization Quality of Life − Brief questionnaire (WHOQOL‐BREF) is a 26‐question quality‐of‐life assessment producing four domain scores: physical health, psychological health, social relationships and environmental, plus a single quality of life question and domain average [16]. The WHOQOL‐BREF psychological score was used to compare to the ATOP psychological health item score.

#### 2.3.4 Physical Health

Two gold standard comparators were used to compare to the ATOP physical health item: the WHOQOL‐BREF physical health domain scores and the Patient Health Questionnaire 15‐Item Somatic Symptom Severity Scale [17]. The Patient Health Questionnaire comprises 15 somatic symptom items, scored from 0 (*not bothered at all*) to 2 (*bothered a lot*) and provides a continuous measure of somatic symptom severity.

#### 2.3.5 Quality of Life

The Personal Wellbeing Index uses nine questions to assess subjective well‐being and demonstrates good construct and convergent validity [18]. The total Personal Wellbeing Index score was compared with the ATOP quality of life item. The first question on the scale, ‘How satisfied are you with your life as a whole?’ was used as an additional comparator. The WHOQOL‐BREF social relationships and environmental domains, the single quality of life question and domain average were also used for comparison to ATOP quality of life.

#### 2.3.6 Recent Arrest

The OTI crime ‘been arrested’ question was used to compare to the ATOP arrested item.

#### 2.3.7 Violence to Another

The OTI violent crime sub‐scale was used to compare to the ATOP violence to someone else item.

#### 2.3.8 Excluded Items

ATOP items for which concurrent validity was not examined as no suitable comparator measures were available are: number of days work and study, care for children and experience of violence.

### 2.4 Data Management and Statistical Analysis

Data were extracted from the LiMA REDCap research data management system [19] and statistical analyses were conducted using IBM SPSS Statistics (Version 25). Continuous data were analysed using Pearson's correlation coefficient and dichotomous data were analysed using Fleiss's *κ*. Agreement was rated as strong where measures were 0.50 or more, moderate where agreement was between 0.30 and 0.49, and weak where under 0.30 [20].

## 3 Results

Data were available for 118 participants (2018–2020); mean age of 39 years (standard deviation [SD] 9 years), 60% men (*n* = 71), 40% women (*n* = 61). All were cisgender, two (2%) reported intersex characteristics. Just over three‐quarters were heterosexual (*n* = 90, 76%). Thirteen percent (*n* = 11) of the sample were Aboriginal and/or Torres Strait Islander. Approximately half (*n* = 60, 51%) reported highest education of Year 10 level or below, 17% (*n* = 20) to Year 12 level and 33% (*n* = 38) had a trade, technical qualification or degree. Most reported government benefit as their main income (57%, *n* = 67), and 30% (*n* = 35) were employed, nine (8%) reported other income and seven (6%) had no income. The majority of the sample were in rental accommodation, with 29 (25%) in public rental and 43 (36%) in private rental. Twenty‐three (20%) owned their accommodation, and 23 (20%) were recorded as other housing. Just over half (53%) of participants were from the three Sydney sites (27, 22 and 14 participants), 21 (18%) from the Adelaide site, 21 (18%) from the Newcastle site and 13 (11%) from Melbourne. Table 1 (continuous items) and Table 2 (dichotomous items) provide the concurrent validity reliability statistics for the ATOP. Days of heroin use and days of other opioid use were endorsed by too few participants for adequate assessment. Violence to another was unable to be assessed as no participants endorsed the comparator question (OTI violent crime). All other ATOP items achieved strong or moderate concurrent validity, apart from benzodiazepines use days.

**TABLE 1:** Concurrent validity of continuous ATOP items.

ATOP item	Comparator	ATOP mean (SD)	Comparator, mean (SD)	PCC (95% CI)
Alcohol days used	TLFB	6 (8)	6 (8)	0.96 (0.93–0.98)^f^
Cannabis days used	TLFB	12 (11)	12 (11)	0.96 (0.93–0.99)^f^
Amphetamines days used	TLFB	23 (7)	24 (5)	0.66 (0.47–0.86)^f^
Benzodiazepines days used	TLFB	4 (4)	2 (6)	−0.07 (−0.50–0.18)^d^
Heroin days used	TLFB	1 (1)	1 (1)	0 (−)^a^
Other opioids days used	TLFB	7 (9)	2 (1)	−0.65 (−)^a^
Cocaine days used	TLFB	1 (1)	2 (3)	0.68 (−0.87–0.95)^f^
Injected drugs, days	OTI HIV risk question 1	19 (8)	3 (1)^b^	0.58 (0.28–0.82)^f^
Psychological health	DASS score	6 (2)	42 (23)	−0.46^c^ (−0.58 to −0.31)^e^
Psychological health	WHOQOL‐BREF psychological domain	5 (2)	12 (3)	0.61 (0.47–0.73)^f^
Physical health	PHQ‐15	6 (2)	7 (4)	−0.30^c^ (−0.44 to −0.14)^e^
Physical health	WHOQOL‐BREF physical domain	6 (2)	13 (3)	0.49 (0.31–0.65)^f^
Quality of life	PWI Q1 score	5 (2)	5 (2)	0.52 (0.37–0.64)^f^
Quality of life	PWI total score	5 (2)	5 (2)	0.60 (0.47–0.72)^f^
Quality of life	WHOQOL‐BREF question 1 score	6 (2)	3 (1)	0.56 (0.38–0.72)^f^
Quality of life	WHOQOL‐BREF domain average	6 (2)	33 (14)	0.50 (0.34–0.63)^f^
Quality of life	WHOQOL‐BREF social domain	6 (2)	11 (4)	0.35 (0.18–0.50)^e^
Quality of life	WHOQOL‐BREF environmental domain	6 (2)	13 (3)	0.48 (0.32–0.62)^e^

Abbreviations: ATOP, Australian Treatment Outcomes Profile; DASS, Depression Anxiety and Stress Scale; OTI, Opiate Treatment Index; PCC, Pearson's Correlation Coefficient; PHQ‐15, Patient Health Questionnaire 15‐Item Somatic Symptom Severity Scale; PWI, Personal Wellbeing Index; SD, standard deviation; TLFB, Timeline Followback; WHOQOL‐BREF, World Health Organization Quality of Life − Brief.

^a^ Too small a sample for calculation of confidence intervals.

^b^ Five‐point scale from none to three times/day.

^c^ Negative correlation due to reverse scoring of comparator scales.

^d^ Weak agreement (PCC < 0.30).

^e^ Moderate agreement (0.30 ≤ PCC ≤ 0.49).

^f^ Strong agreement (PCC ≥ 0.50).

**TABLE 2:** Concurrent validity of dichotomous ATOP variables.

ATOP	Comparator		ATOP		% agreement	*κ* (95% CI)
			No, *n*	Yes, *n*		
Alcohol	TLFB	No, *n*	50	9	90%	0.80 (0.68–0.90)^c^
		Yes, *n*	3	56		
Cannabis	TLFB	No, *n*	75	2	97%	0.92 (0.84–0.98)^c^
		Yes, *n*	2	38		
Amphetamines	TLFB	No, *n*	0	0	99%	0.00^a^
		Yes, *n*	1	117		
Benzodiazepines	TLFB	No, *n*	91	10	90%	0.66 (0.46–0.83)^c^
		Yes, *n*	1	14		
Heroin	TLFB	No, *n*	110	2	97%	0.58 (−0.01 to 0.90)^c^
		Yes, *n*	2	3		
Other opioids	TLFB	No, *n*	109	2	97%	0.76 (0.39–1.00)^c^
		Yes, *n*	1	5		
Cocaine	TLFB	No, *n*	104	2	97%	0.80 (0.56–0.96)^c^
		Yes, *n*	2	9		
Tobacco use	TLFB	No, *n*	23	1	97%	0.90 (0.78–0.98)^c^
		Yes, *n*	3	89		
Injected drugs	OTI HIV risk	No, *n*	61	1	99%	0.98 (0.95–1.00)^c^
		Yes, *n*	0	52		
Injected with used equipment	OTI HIV risk	No, *n*	109	1	98%	0.49 (−0.02 to 1.00)^b^
		Yes, *n*	1	1		
Arrested	OTI arrest	No, *n*	109	2	97%	0.65 (0.20–0.92)^c^
		Yes, *n*	2	4		

Abbreviations: ATOP, Australian Treatment Outcomes Profile; CI, confidence interval; OTI, Opiate Treatment Index.

^a^ Unable to be tested as all participants were required to have used amphetamines in the previous 28 days.

^b^ Moderate agreement (0.30 ≤ *κ* ≤ 0.49).

^c^ Strong agreement (*κ* ≥ 0.50).

## 4 Discussion

This study provides the first validation of the ATOP tool for a treatment‐seeking population of people with methamphetamine dependence. Nearly all ATOP items reached moderate or strong agreement for concurrent validity. This expands the range for validated assessment tools for clinicians for use with this population and enhances the consistency of outcome monitoring. The use of standardised tools within routine practice also supports quality improvement, evaluation and research activities across differing services and settings.

A number of limitations should be noted. The sample is drawn from a clinical trial cohort with 14 days or greater of methamphetamine use in specialist clinics, and so findings may not generalise to less frequent use or to other treatment settings. The data were also collected prior to the COVID‐19 pandemic, and so any changes to substance use patterns that have occurred since then may limit application. It should be noted that this study examined methamphetamine use rather than the broader category of amphetamine type stimulants assessed with the ATOP. Nearly all treatment seeking for amphetamine type stimulants in Australia, however, is in relation to methamphetamine [8]. Although benzodiazepine use days was endorsed by a sufficient number of participants, it failed to reach adequate concurrent validity. This is likely due to the ATOP collecting all benzodiazepine use (prescribed and non‐prescribed), whereas the TLFB used in the LiMA study collected non‐prescribed use only, and so further testing is required to establish validity for this item.

Future work on the ATOP could include broadening validity for this population outside of clinical trial cohort samples, as well as establishing inter‐rater reliability. Establishing the validity of the instrument for self‐administration in online and paper versions and for remote clinician administration may also be useful given the growth of telehealth and digital interventions.

## 5 Conclusions

The ATOP can be confidently used for assessment of substance use, health and well‐being in clinical populations presenting for treatment of their methamphetamine use. The ATOP has now been validated for use with the most common substances (alcohol, methamphetamines, cannabis and opioids) for which clients of Australian alcohol and other drug services seek treatment.

## Author Contributions

Each author certifies that their contribution to this work meets the standards of the International Committee of Medical Journal Editors.

## Funding

The LiMA study was supported by the National Health and Medical Research Council (APP1109466), St Vincent's Inclusive Health Program and the Curran Foundation.

## Disclosure

The National Centre for Clinical Research on Emerging Drugs receives funding from the Australian Commonwealth Department of Health, Aging and Disability.

## Conflicts of Interest

N.L. has received honoraria for presenting professional education at international symposia from Camurus AB and Indivior. N.L.'s employer has received funding from Camurus AB for unrelated research. The other authors declare no conflicts of interest.

## Data Availability

The data that support the findings of this study are available on request from the corresponding author. The data are not publicly available due to privacy or ethical restrictions.
